# Controllability Attribution as a Mediator in the Effect of Mindset on Achievement Goal Adoption Following Failure

**DOI:** 10.3389/fpsyg.2019.02943

**Published:** 2020-01-15

**Authors:** Juyeon Song, Sung-il Kim, Mimi Bong

**Affiliations:** ^1^Department of Education, Korea National University of Education, Chungbuk, South Korea; ^2^Department of Education, Brain and Motivation Research Institute (bMRI), Korea University, Seoul, South Korea

**Keywords:** growth mindset, attribution, controllability, achievement goal, academic failure

## Abstract

The purpose of this study was to examine the effect of a growth mindset on achievement goal adoption in the face of failure. We also sought to investigate the mediating role of controllability attribution in order to understand the underlying process behind the effect of mindset on achievement goal adoption following failure. One hundred and seventy-two 4^th^ and 5^th^ grade students participated in an experimental task. In the manipulation phase, in related to the experimental task, 71 participants were provided with growth mindset-based information, and the other 101 were provided with fixed mindset-based information. After completing the experimental task on a computer, all participants were informed that they had failed the task. The participants then responded to controllability attribution and achievement goal scales. We empirically demonstrated that a growth mindset had a positive influence on mastery goal adoption, while it did not predict performance goal adoption. We also found that controllability attribution had a full mediation effect on the relationship between the presence of a growth mindset and mastery goal adoption; this finding implies that a key element in promoting the adoption of mastery goals following failure is attributing the failure to controllable causes, a belief which arises from a stronger growth mindset.

## Introduction

All students experience failure in the learning process, and failure experiences can intensify the fear of failure, cause anxiety, or reduce perceived competence (Miller and Hom, 1990; Elliot, 1999). Failure experiences are therefore a major reason for students becoming less motivated. Nevertheless, the impact of failure in a task is not the same for every student; some may continue to engage in the task, whereas others may abandon the task or choose extremely easy or difficult levels in subsequent tasks to mask their true ability (Dweck and Leggett, 1988).

Several motivational theories, such as mindset theory, attribution theory, and achievement goal theory are linked in that they all help to understand motivational changes and behavior in students, especially in the face of failure (Diener and Dweck, 1978; Elliott and Dweck, 1988; Mueller and Dweck, 1998; Weiner, 2010; Dweck, 2012). However, with the exception of some early studies (Diener and Dweck, 1978; Elliott and Dweck, 1988), there has been little empirical evidence for a relationship between these motivational constructs in failure situations. Therefore, the purpose of this study was to examine how students’ mindsets and attribution beliefs affect their motivational reaction to failure, especially in relation to achievement goal adoption.

### Theoretical Background

#### Mindset and Achievement Goals in the Face of Failure

Achievement goals are the aims or reasons for achievement behavior (Midgley et al., 2000). In learning, students’ behavioral styles can vary according to the type of achievement goal that is pursued. Two qualitatively different types of achievement goals are mastery goals and performance goals. Mastery goals focus on developing one’s own ability, while performance goals focus on proving that own’s ability is superior to others’ one (Nicholls, 1984; Dweck, 1986). Students who pursue mastery goals are more likely to seek challenges, have intrinsic motivation, and demonstrate greater persistence following failure, whereas those who pursue performance goals are more likely to avoid challenges and be vulnerable to failure (Dweck and Leggett, 1988; Nicholls, 1989).

Early experimental studies demonstrated that two types of mindset about the nature of ability (used interchangeably with the theory of intelligence) have a major impact on achievement goal adoption (Dweck and Leggett, 1988). In past experiments, a growth mindset (also known as an incremental belief) or a fixed mindset (also known as an entity belief) was instilled in participants through the reading of a passage that described either a growth or fixed mindset. The growth mindset passages tend to state that an individual’s ability can be improved, whereas the fixed mindset passages tend to argue that intelligence and ability are fixed traits (e.g., Bergen, 1991; Hong et al., 1999). This manipulation of mindset can influence students’ achievement goal adoption (Dweck and Leggett, 1988). When students have a growth mindset, they are more likely to focus on improving their malleable ability (i.e., mastery goals), while students with a fixed mindset are more likely to focus on demonstrating or hiding their fixed ability (i.e., performance goals).

According to the trichotomous approach to achievement goals (Elliot and Church, 1997), performance goals can be further divided into performance-approach and performance-avoidance goals based on competence valence (i.e., competence beliefs such as self-efficacy). Performance-approach goals focus on demonstrating a fixed superior ability, whereas performance-avoidance goals focus on hiding a fixed inferior ability. According to a meta-analysis study based on survey studies (Burnette et al., 2013), a growth mindset was positively related with mastery goals, which was consistent with earlier experimental studies. As expected, both performance-approach and performance-avoidance goals had a negative relationship with a growth mindset, with this negative relationship stronger for performance-avoidance goals.

The same meta-analysis study by Burnette et al. (2013) found that the relationship between a growth mindset and achievement goals becomes stronger in ego-threating situations, which are defined as any events or situations that have an unfavorable effect on the self. Considering that a failure experience is a common ego-threating event in academic settings, a stronger relationship between a growth mindset and achievement goals can be found following failure. In this regard, compared to students who received higher scores, those who received lower scores had a stronger relationship between a growth mindset and mastery goals (Aditomo, 2015).

However, more empirical evidence is required to clearly understand the relationship between a fixed mindset and the adoption of performance-approach and performance-avoidance goals. A recent experimental manipulation of mindset found that participants in a growth mindset condition were more likely to pursue mastery goals compared to those in a fixed mindset condition (Lou and Noels, 2016). Unlike mastery goals, in pursuing performance-approach and avoidance goals, there was no difference between the two conditions, meaning that the mindset manipulation did not significantly affect performance goal adoption. The role of mindset in predicting three achievement goals was mainly proved by survey research, but little was proved by experimental research. Therefore, additional experimental studies are needed to verify whether manipulating mindset can influence the adoption of performance-approach and performance-avoidance goals, especially following failure.

#### Controllability Attribution as a Mediator

The evidence accumulated so far clearly indicates that a growth mindset plays a pivotal role in adopting mastery goals. Nevertheless, evidence is not sufficient to explain how a growth mindset positively affects achievement goal adoption following failure. Attribution has been widely identified as an important psychological mechanism that can explain the adaptive function of a growth mindset in the face of failure (Dweck and Reppucci, 1973; Dweck, 1975; Diener and Dweck, 1978; Hong et al., 1999). After experiencing failure, students interpret this failure in accordance with their beliefs, and this subjective interpretation affects their feelings, perceptions, and behavior (Weiner, 2010). In this situation, a student’s mindset is a belief that can have a major impact on his/her own understanding of failure (Dweck, 2000). For students who have a growth mindset, a failure indicates the need to put more effort into the task to improve their intelligence or their ability to perform well on the task, and thus they are more likely to attribute their failure to insufficient effort. In contrast, to students who have a fixed mindset, a failure represents low intelligence or ability, and thus they are more likely to attribute their failure to their ability.

Previous survey and experimental research have supported the relationship between the presence of a growth mindset and effort attribution. For example, when students are asked to find a cause of their mid-term exam results, students who had a growth mindset tended to attribute their exam score to their effort (Aditomo, 2015). This tendency was found to be stronger among low achievers. An experiment study which manipulated growth and fixed mindsets also yielded the same results (Hong et al., 1999). After receiving negative feedback, participants with a fixed mindset attributed their performance more to ability than to effort. A comparison between participants with a growth mindset and those with a fixed mindset revealed that the growth mindset group had weaker ability attribution and stronger effort attribution than the fixed mindset group.

In such experimental studies, however, there is a need to pay attention to the scores of effort and ability within the growth mindset group. Participants in the growth mindset condition attributed their failure to their ability as much as to their effort (Hong et al., 1999). For these participants who had a growth mindset, however, neither effort nor ability attribution itself had a significant impact on their motivation or behavior following failure. Because, the impact of ability attribution on the perceptions, emotions, and behavior following failure can differ depending on the growth mindset. In classic attribution theory, ability is seen as an uncontrollable cause (e.g., Weiner et al., 1972). However, if students have a growth mindset, they will not see ability as uncontrollable even though they can attribute their failure to their ability. Only students who have a fixed mindset will view their fixed ability as uncontrollable.

Attribution theory also assumes that three characteristics of causes (i.e., causal dimensions) determine students’ responses after failure (Weiner, 2010): locus (whether a cause is something inside or outside the attributor), stability (whether a cause is something stable or variable over time), and controllability (whether a cause is something controllable or not). These dimensions are distinct from actual causes of failure themselves (e.g., effort, ability, task difficulty, and luck). Although both stability and controllability may vary according to a student’s mindset, Dweck and Leggett (1988) considered controllability as the key to explaining the positive function of mindset in failure situations. That is, the growth mindset increases the perception of controllability of a cause of failure, and this can play a major role in helping students to overcome difficulty or failure.

However, few studies have directly examined the relationship between students’ mindset and controllability attribution. In particular, there is a lack of research examining the motivational responses of students in failure situations, and in those studies that have done so, controllability as a causal dimension has not been directly measured; rather, perceived causes of failure *per se* (e.g., effort and ability) have been assessed (e.g., Hong et al., 1999; Aditomo, 2015). However, as described above, in order to accurately demonstrate the function of a growth mindset in a failure situation, it is necessary to directly measure controllability attribution rather than the causes of failure. In addition, most previous studies used hypothetical scenarios or short descriptions (e.g., “Rate how important you think each of the following factors was in determining the grades you received last semester”; Robins and Pals, 2002) to measure attribution, rather than assessing attribution after experiencing actual failure. Therefore, in this study, an experiment was designed to directly measure controllability attribution after the participants had failed in a task. This would provide empirical evidence that could be used to explain the underlying mechanism of the relationship between a growth mindset and achievement goal adoption.

### Present Study

A growth mindset can be instilled in individuals by having them listen to simple instructions or read text (Chiu et al., 1997; Good et al., 2003; see Yeager and Dweck, 2012 for a review). Therefore, in this study, we attempted to investigate the effect of mindset on achievement goal adoption in failure situations by manipulating the growth or fixed mindset of students using a newspaper article written for the purposes of the study. In particular, we tried to verify the effect of mindsets after controlling for self-efficacy and anxiety, which can affect motivation in failure situations (Elliot and Church, 1997; Elliot and McGregor, 1999). Furthermore, we sought to explain the psychological mechanisms behind the effect of mindsets on achievement goal adoption by assessing controllability attribution.

First, we hypothesized that students in the growth mindset condition would be more likely to pursue mastery goals than those in the fixed mindset condition. In contrast, participants with a fixed mindset would be more likely to pursue performance goals, especially performance-avoidance goals, because they would have received feedback on their failure. Second, we believed that the positive function of a growth mindset in this situation would be explained by controllability attribution. That is, controllability attribution would mediate the effect of a growth mindset on mastery goal adoption.

## Materials and Methods

### Participants

The participants were 172 fourth and fifth graders from seven classes in four elementary schools in a metropolitan city in Korea. The ages of the participants ranged from 10 to 12 at the time of the experiment, based on the official school enrollment age in Korea. Each class was randomly assigned to one of two conditions; three classes (71 students, 37 boys and 34 girls) were assigned to the growth mindset group, and four classes (101 students, 50 boys and 51 girls) were assigned to the fixed mindset group.

The schools participating in this study were provided information about the study and could opt out if desired. All teachers and parents also received all information about this study from school announcements. None of the parents doubted or opposed this research; however, we did not receive written consent from the parents. All participants and their parents were given an opt-out option, and participants voluntarily participated in the research. Participants were informed that their responses would be used for research purposes only. After the experiment had been fully completed, the participants were thoroughly briefed on the content of the study. Although this study was not reviewed and approved by an institutional review board (ethics committee) before the study began because an ethics approval was not mandatory as per applicable institutional and national guidelines and regulations at the time, the experiment was conducted without violating research ethics.

### Procedures and Required Task

Supplementary Figure S1 shows the procedures of the experiment. Before the experiment, the participants completed an anxiety questionnaire in their classroom, because anxiety is a personality variable that can influence motivation following failure. The participants then moved to a computer room at their elementary school and sat at a computer that had been assigned to them. The experimenter explained that the participants would learn what information processing intelligence was and would then complete a task that required information processing intelligence. The experimenter also explained how to complete the information processing task and the criterion for success, which was having the correct answer in more than 8 or more of the 15 trials. Each participant then practiced the information-processing task for three trials. After finishing this practice, the participants completed the self-efficacy questionnaire.

To manipulate the participants’ mindset, they were given a persuasive newspaper article with a colorful graph. The article for the growth mindset condition included information such as “……The Educational Development Institute reported that, after testing the information processing intelligence of elementary school students, they have come to the conclusion that anyone can increase their information processing intelligence through education……It seems as though information processing intelligence does not require inborn intelligence.” The article for the fixed mindset condition included information such as “……The Educational Development Institute reported that, after testing the information processing intelligence of elementary school students, they have come to the conclusion that it is impossible to increase information processing. …… information processing intelligence is inborn.” These articles were written based on previous studies in which researchers successfully instilled a growth mindset using newspaper articles (Hong et al., 1999; Dweck, 2000; Cury et al., 2006). The participants read the newspaper article while listening to an explanation of the article by the experimenter and then completed a questionnaire on mindset to determine whether the manipulation had been successful.

Following this, the participants performed the experimental task, which consisted of 15 trials. As soon as they completed the task, they were given the following bogus failure feedback regardless of their actual performance: “You failed this task.” Participants were then asked to choose whether they thought the cause of their failure was a lack of ability or lack of effort. Subsequently, they responded to a controllability attribution question, asking if the cause of failure they thought was controllable. Finally, prior to the achievement goal survey, participants were informed that there would be a supplementary task on a computer. They then completed the achievement goal questionnaire, having in mind the upcoming task. However, participants did not actually perform the additional task, but instead received a debriefing about the purpose of the experiment as soon as the survey was completed.

Each trial of the information-processing task (see Supplementary Figure S2) involved judging as quickly and accurately as possible whether the number of target stimuli (the letter “T”) briefly presented on the screen was higher or lower than the number of distracting stimuli (the letter “F”) over a period of 4 s. This task seemed suitable for testing information processing intelligence because the participants were informed that information processing intelligence is the ability to quickly and accurately handle surrounding information. In addition, this task did not allow for the number of letters to be counted exactly. Due to the short presentation time, it was difficult for the participants to be confident that they had found the correct answer each time. Therefore, the bogus feedback was plausible for the participants, and they did not doubt it.

### Measures

All scales were rated on a five-point Likert scale ranging from 1 (strongly disagree) to 5 (strongly agree). Growth and fixed mindset, self-efficacy, and achievement goal items were revised from previous scales to focus on the given information-processing task.

Two growth mindset items and two fixed mindset items were adapted from Dweck’s (2000) scale to ensure that the manipulation of student mindset would be successful. The growth mindset items (ω = 0.89) included “Information processing intelligence can be improved through education” and “The score in an information processing task can be improved through education.” The fixed mindset items (ω = 0.67) included “Information processing intelligence is an inborn trait” and “A high or low level of information processing intelligence is fixed.”

We used seven self-efficacy items (sample item: “I believe I will succeed in this information processing task”) adopted from the self-efficacy subscale of the Motivated Strategies for Learning Questionnaire (MSLQ; Pintrich and De Groot, 1990). This scale exhibited sufficient reliability (ω = 0.90). To measure anxiety, we used four items adopted from the behavioral inhibition system scale (Poythress et al., 2008). A sample item is “I worry about making mistakes.” The omega coefficient was 0.63.

For controllability attribution, two items were adapted from Russell’s (1982) scale to fit the experimental situation. After the participants had attributed their failure to either inferior ability or insufficient effort, they scored the controllability of what they had chosen. Controllability attribution items included “Abilities/efforts can change if I try to change them” and “Ability/effort is something that I can change.” The scale showed a reliable omega coefficient of 0.80.

Finally, we used five mastery goal items (sample item: “One of my goals in this task is to learn the skills to improve my information processing intelligence as much as I can”; ω = 0.87), five performance-approach goal items (sample item: “One of my goals is to show others that I’m good at this task”; ω = 0.80), and four performance-avoidance goal items (sample item: “One of my goals in this task is to avoid looking like I am having trouble doing this task”; ω = 0.74) from the Patterns of Adaptive Learning Scales (PALS; Midgley et al., 2000).

### Statistical Analysis

First, we confirmed the success of the manipulation and tested the direct effects of mindset on controllability attribution and achievement goals using independent *t*-tests in SPSS 18. Following this, structural equation modeling (SEM) was employed to test the mediating role of controllability attribution in the relationship between mindset and achievement goal adoption after controlling for anxiety and self-efficacy. Chi-square statistics and multiple goodness-of-fit indexes, including the Tucker-Lewis Index (TLI), comparative fit index (CFI), root mean square error of approximation (RMSEA), and standardized root mean square residual (SRMR) were used to evaluate the overall model fit. Coefficients above 0.90 indicated a suitable fit for the CFI and TLI (Hu and Bentler, 1999), values under 0.05 indicated a close approximate fit for the RMSEA and the SRMR, and values between 0.05 and 0.08 represented a reasonable fit (Browne and Cudeck, 1993). To deal with the low percentage of missing values (0.0–4.1%), we used full information maximum likelihood (FIML) estimation, as recommended by Graham (2009). The multilevel structure was not of primary substantive interest in the present study, but students were nested within classes. According to McNeish et al. (2017) recommendations, we applied the design-based correction of standard errors and fit statistics (with type = complex) in Mplus 7.31.

## Results

### Descriptive Statistics and Latent Correlations

Table 1 presents the descriptive statistics, reliability, and latent correlations for the measured variables. The absolute values for the skewness index and the kurtosis index were less than | 0.42| and | 0.62|, respectively, indicating that all the measures produced an approximately normal distribution of scores (Kline, 2011).

**TABLE 1:** Descriptive statistics, reliabilities, and latent correlations.

**Variable**	**1**	**2**	**3**	**4**	**5**	**6**	**7**
(1) Mindset group	−						
(2) Anxiety	–0.20	−					
(3) Self-efficacy	–0.02	0.03	−				
(4) Controllability attribution	0.28^∗∗∗^	0.12	0.28^∗∗∗^	−			
(5) Mastery goal	0.20^∗^	0.20	0.20^∗^	0.53^∗∗∗^	−		
(6) Performance-approach goal	–0.04	0.22^∗∗^	0.51^∗∗∗^	0.28^∗^	0.30^∗^	−	
(7) Performance-avoidance goal	–0.03	0.27^∗∗^	0.28^∗∗^	0.22^∗^	0.29^∗∗^	0.85^∗∗∗^	−
*M*	−	3.29	3.13	3.75	3.71	2.44	2.75
*SD*	−	0.89	0.92	0.98	0.92	0.87	0.94
Skewness	−	–0.36	–0.04	–0.40	–0.42	0.40	0.21
Kurtosis	−	–0.56	–0.25	–0.62	–0.35	–0.05	–0.33
α	−	0.62	0.91	0.81	0.86	0.80	0.74
ω	−	0.71	0.91	0.86	0.89	0.84	0.80

*Mindset group was a dichotomous variable with the growth mindset group coded as 1 and the fixed mindset group coded as −1. ^∗^*p* < 0.05; ^∗∗^*p* < 0.01; ^∗∗∗^*p* < 0.001.*

As expected, anxiety was positively correlated with performance-approach goals (*r* = 0.22, *p* = 0.01) and performance-avoidance goals (*r* = 0.27, *p* = 0.005) but was not significantly correlated with mastery goals. Self-efficacy was positively correlated with all achievement goals. However, the correlation between self-efficacy and performance-approach goals (*r* = 0.51, *p* < 0.001) was noticeably higher than the correlations between self-efficacy and performance-avoidance goals (*r* = 0.28, *p* = 0.005) and mastery goals (*r* = 0.20, *p* = 0.011). Self-efficacy was also positively correlated with controllability attribution (*r* = 0.28, *p* < 0.001), which, in turn, was positively correlated with mastery goals (*r* = 0.53, *p* < 0.001) and performance-approach goals (*r* = 0.28, *p* = 0.02). However, controllability attribution was also positively correlated with performance-avoidance goals (*r* = 0.22, *p* = 0.027). Mastery goals were positively correlated with performance-approach goals (*r* = 0.30, *p* = 0.026) and performance-avoidance goals (*r* = 0.29, *p* = 0.001). Performance-approach and performance-avoidance goals were highly and positively correlated with each other (*r* = 0.85, *p* < 0.001).

### Main Effect of Mindset

As presented in Table 2, the participants in the growth mindset group showed significantly higher growth mindset scores than did those in the fixed mindset group, *t* = −7.83, *p* < 0.001. Similarly, the participants in the fixed mindset group showed significantly higher fixed mindset scores than did those in the growth mindset group, *t* = 4.46, *p* < 0.001. Taken together, these results indicate that the mindset manipulation was successful.

**TABLE 2:** Manipulation check.

	**Growth mindset group (*n* = 101)**		**Fixed mindset group (*n* = 71)**	
**Variable**	***M***	***SD***	***M***	***SD***
Fixed mindset	2.80	1.10	3.57	1.13
Growth mindset	4.27	0.89	2.96	1.30

*Students in the growth mindset group showed significantly higher growth mindset scores than those in the fixed mindset group, *t* = −7.83, *p* < 0.001. Conversely, students in the fixed mindset group showed significantly higher fixed mindset scores than those in the growth mindset group, *t* = 4.46, *p* < 0.001.*

As presented in Table 3, mastery goal scores were significantly higher for the growth mindset group than for the fixed mindset group, *t* = −2.52, *p* < 0.01, while there were no differences in performance-approach and performance-avoidance goal scores. Controllability attribution was also higher for the growth mindset group than the fixed mindset group, *t* = −3.38, *p* < 0.001. We additionally performed a chi-squared test to ascertain if the students in the growth mindset group attributed their failures to lack of effort rather than lack of ability compared to the students in the fixed mindset group. In total, 47.9% of the students in the growth mindset group attributed their failure to their lack of ability, and 47.9% attributed their failure to lack of effort (4.2% = missing data), while 53 and 44% of the students in the fixed mindset group attributed their failure to lack of ability and effort, respectively (2% = missing data). The difference between the two groups was not significant (*p* = 0.637).

**TABLE 3:** Direct effects by group.

	**Growth mindset group (*n* = 71)**		**Fixed mindset group (*n* = 101)**		***t***
**Variable**	***M***	***SD***	***M***	***SD***	
Mastery goal	3.92	0.84	3.56	0.96	−2.52^∗^
Performance-approach goal	2.41	0.89	2.46	0.86	0.44
Performance-avoidance goal	2.73	0.88	2.77	0.99	0.25
Controllability attribution	4.06	0.91	3.47	1.25	−3.38^∗∗∗^

*^∗^*p* < 0.05; ^∗∗∗^*p* < 0.001.*

### Mediation Effect by Controllability Attribution

**FIGURE 1:**
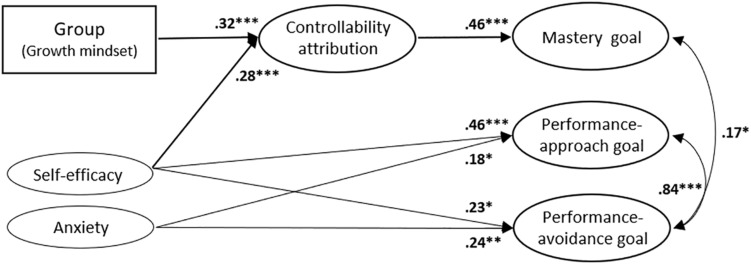
The mediating role of controllability attribution. In a dummy variable called “Group (Growth mindset),” the growth mindset group was coded as 1, and the fixed mindset group was coded as –1. Bold paths indicate the significant indirect paths. We examined three correlations between exogenous variables, three correlations between disturbance terms of the all the dependent variables, and direct and indirect paths of group and control variables for achievement goals, but only statistically significant paths are shown in this figure. For clarity, indicators, measurement errors, and disturbance terms are also not presented. ^∗^*p* < 0.05; ^∗∗^*p* < 0.01; ^∗∗∗^*p* < 0.001.

To test the mediating role of controllability attribution, we tested a hypothetical model in which group (i.e., a dummy variable for which a growth mindset was coded as 1 and a fixed mindset was coded as −1) was set as an exogenous variable, achievement goals were set as outcome variables, and controllability attribution was set as a mediator, while controlling for self-efficacy and anxiety (see Figure 1). This model demonstrated an acceptable fit for the empirical data, χ^2^(330, *N* = 172) = 478.434, *p* < 0.001 (CFI = 0.914, TLI = 0.902, RMSEA = 0.051, SRMR = 0.061).

As hypothesized, group positively predicted controllability attribution (β = 0.32, *p* < 0.001), indicating that students in the growth mindset group were more likely to perceive the cause of their failure to be more controllable than those in the fixed mindset group. Controllability attribution positively predicted mastery goals (β = 0.46, *p* < 0.001). The indirect effect from group to mastery goals via controllability was thus significantly positive (β = 0.13, *p* = 0.011), but the direct effect was not (β = 0.09, *p* = 0.324). This means that the effect of group on mastery goals was fully mediated by controllability attribution. In addition, self-efficacy positively predicted controllability attribution (β = 0.28, *p* < 0.001). Controllability attribution also mediated the relationship between self-efficacy and mastery goals (β = 0.11, *p* = 0.010).

However, group did not significantly predict performance-approach and performance-avoidance goals. Rather, self-efficacy positively predicted both performance-approach goals (β = 0.46, *p* < 0.001) and performance-avoidance goals (β = 0.23, *p* = 0.023). Anxiety also positively predicted both performance-approach goals (β = 0.18, *p* = 0.022) and performance-avoidance goals (β = 0.24, *p* < 0.003).

## Discussion

Failure is a critical experience that affects achievement goal adoption (Nicholls, 1984). The effect of failure experience, however, is not the same for all students, and it can differ depending on the factors that the students attribute the failure to Weiner (2010). We thus examined the relationships between mindset, attribution, and achievement goal adoption in the face of failure. We reaffirmed the positive effect of a growth mindset on mastery goal adoption, and we found that controllability attribution mediated the effect of a growth mindset on mastery goal adoption. However, the effects of mindset on the pursuit of performance-approach and performance-avoidance goals were not significant. Rather, self-efficacy and anxiety directly predicted performance-approach and performance-avoidance goals.

### Controllability Attribution as a Mechanism Underlying Mastery Goal Adoption

Consistent with previous research, a growth mindset was found to have a positive influence on mastery goal adoption in the face of failure (Dweck and Leggett, 1988; Aditomo, 2015). We further found that this effect can be fully mediated by controllability attribution. Even though all of the students experienced the same type of failure in the task, the presence of a growth mindset affected their attribution. The stronger the growth mindset, the more the students recognized that they could control the cause of failure, regardless of effort or ability. Those students thus pursued mastery goals despite their experience of failure. Self-efficacy played the same role as a growth mindset, and this was also fully mediated by controllability attribution.

Although numerous researchers have asserted that attribution is one of the main explanations for different patterns of mastery goal adoption in the learning process, especially in failure situations (Dweck and Reppucci, 1973; Diener and Dweck, 1978; Hong et al., 1999), few studies have examined the role of attribution in real situations. In particular, the role of the causal dimensions of attribution, such as controllability, stability, and locus have been neglected in past research, even though causal dimensions have more implications for understanding motivational behavior than the causes themselves (Weiner, 2010). In this study, perceived controllability, one of the causal dimensions, differed between the growth mindset and fixed mindset groups, and played a mediating role in the relationship between growth mindset and mastery goal adoption. The number of effort or ability attribution however did not differ between groups.

The attributional dimension of failure can be a proximal predictor of achievement strivings such as achievement goal adoption (Weiner, 2010). Our findings showed that students were more likely to pursue mastery goals when they attributed their failure to controllable factors. In this respect, the present study expands the understanding of the role of attribution as a psychological mechanism in achievement goal adoption. Our findings also provide practical guidance for teachers in helping students to pursue mastery goals, especially when students experience failure with a new task. For example, teachers can help students to pursue mastery goals by attributing their failure to controllable factors, which is possible when students have a growth mindset and high self-efficacy.

### Performance Goal Adoption in the Face of Failure

Students’ mindset is correlated with performance-approach and performance-avoidance goals (Burnette et al., 2013). However, consistent with the results of a recent experiment (Lou and Noels, 2016), the simple manipulation of a fixed mindset did not have a significant impact on either performance-approach or performance-avoidance goal adoption in the present study. Rather, performance goal adoption was directly affected by self-efficacy and anxiety in the face of failure. Consistent with previous research, anxiety was a positive predictor of not only performance-avoidance goals but also performance-approach goals (Elliot and McGregor, 1999). However, unexpectedly, self-efficacy positively predicted both performance-approach and performance-avoidance goals.

This unexpected result may have derived from the strong association between the two types of performance goals (*r* = 0.85), which may have emerged due to uncertainty about an ability belief. The experimental task in the present study was novel to the participants, and the participants may have been sensitized to failure even though they may have been highly confident with the task before experiencing failure. In this situation, it was difficult for them to judge their ability accurately and confidently. Therefore, students who adopted performance-approach goals to demonstrate their superior ability also tended to pursue performance-avoidance goals to conceal their inferior ability at the same time after experiencing failure.

Several researchers have also been interested in the reasons for the high correlation between performance-approach and performance-avoidance goals (Law et al., 2012; Bong et al., 2013). Nicholls (1984) suggested that repeated failure may lead students to be certain about their lack of ability, thereby leading them to pursue the goal of concealing their low ability rather than the goal of demonstrating their high ability. Therefore, if uncertainty about ability disappears due to repeated failure, the correlation between performance-approach and performance-avoidance goals may weaken. In addition, the change in self-efficacy following repeated failure is likely to negatively predict performance-avoidance goals.

Furthermore, perceived uncertainty about competence due to the experience of failure could threaten the self-worth of a student, which may further explain the high correlation between the two performance goals and the positive path from self-efficacy and performance-avoidance goals. The failure experience may have been perceived as more threatening to participants who initially had high self-efficacy on the task. To protect their self-worth, therefore, they might pursue both performance-avoidance and performance-approach goals after experiencing failure once. Bong et al. (2013) also found a high correlation between performance-approach and performance-avoidance goals and argued that the strong association might be because the motive to protect one’s self-worth becomes stronger in a threatening environment.

Self-worth threats can be further exacerbated when performance goals are measured as performance ability goals, as defined in the initial achievement goal theory (Dweck and Leggett, 1988). In recent years, there has been debate about the concept of performance goals (Grant and Dweck, 2003; Hulleman et al., 2010). Performance goals have been classified into ability performance goals, which involve efforts to reveal or hide fixed abilities, and normative performance goals, which involve efforts to do better or worse than others. A recent experimental study showed that students experience greater anxiety in an experimental context when manipulated to have ability performance goals than normative performance goals (Chung et al., 2019). Grant and Dweck (2003) also found that, compared to students who pursued mastery goals, students who pursued ability performance goals were more likely to lose their self-worth, attribute their failure to low ability, and ruminate on their failure experiences when they read about a failure experience and imagined it happening to them (Grant and Dweck, 2003). In the present study, performance goals were measured with PALS items (Midgley et al., 2000) that represented ability performance goals (i.e., those that seek to validate ability), as in experiment studies based on early achievement goal theory. Participants who have performance-approach goals but experience a failure might feel anxious, and they may aim not only to validate their ability but also to conceal their possible inferiority in order to protect their self-worth.

### Limitations and Directions for Future Research

First, the present experimental studies have revealed the relationship between mindset and achievement goal adoption, but the effects need to be re-verified with various samples and in diverse contexts. Second, there is still a need for further validation to more fully understand the psychological mechanisms behind the impact of mindset on achievement goal adoption. This study was limited in measuring the stability and locus dimensions of attribution. The present study investigated the role of controllability because it is the concept most closely related to the growth mindset theory (Dweck and Leggett, 1988). This study demonstrated that controllability attribution played a mediating role as a psychological mechanism in mastery goal adoption following failure. Further investigation of the role of stability and locus will enable the psychological mechanisms in achievement goal adoption to be fully understood. In addition, because the measurement of causal dimensions in elementary and early adolescents have not been systematically validated, the validation of these measurements will contribute to strengthening attribution research. Fourth, this study did not provide results based on objective behavioral data such as task choice and task performance. Future research needs to go one step further and examine behavioral patterns varying depending on achievement goal adoption following failure. Last, this study tested the effect of only one failure experience, and it is necessary to examine the effects of continuous failure on academic motivation.

## Conclusion

The present study has theoretical and educational contributions. We found that in the face of failure, the positive effect of a growth mindset on mastery goal adoption can be fully mediated by controllability attribution. This finding deepens the understanding of the psychological processes involved in achievement goal adoption in failure situations. Even after experiencing failure, attributing failure to controllable factors is critical to adopting mastery goals, which predict adaptive outcomes in learning. This finding will thus be useful for encouraging students to maintain their effort on new tasks even in the face of failure.

## Data Availability Statement

The datasets generated for this study are available on request to the corresponding author.

## Ethics Statement

The schools participating in this study were given information about the study and were allowed to opt out if desired. Teachers and parents also received information about this study from school announcements, with none questioning the study. All participants voluntarily participated in the research. They were informed that their responses would be used for research purposes only. After the experiment had been fully completed, the participants were thoroughly briefed on the content of the study. Although this study was not reviewed and approved by an institutional review board (ethics committee) before the study began because IRB approval was not mandatory at the time of the experiment, the experiment was conducted in accordance with the ethical guidelines.

## Author Contributions

JS, SK, and MB designed the research and wrote the manuscript. JS collected and analyzed the data.

## Conflict of Interest

The authors declare that the research was conducted in the absence of any commercial or financial relationships that could be construed as a potential conflict of interest.
