# Supplementary material for: Controllability Attribution as a Mediator in the Effect of Mindset on Achievement Goal Adoption Following Failure
Source: Front Psychol. 2020 Jan 15;10:2943. doi: 10.3389/fpsyg.2019.02943 (PMC6974511; doi:10.3389/fpsyg.2019.02943)
Supplement: Supplementary file 1 [file Data_Sheet_1.PDF]

Supplementary Material

1 Supplementary figures

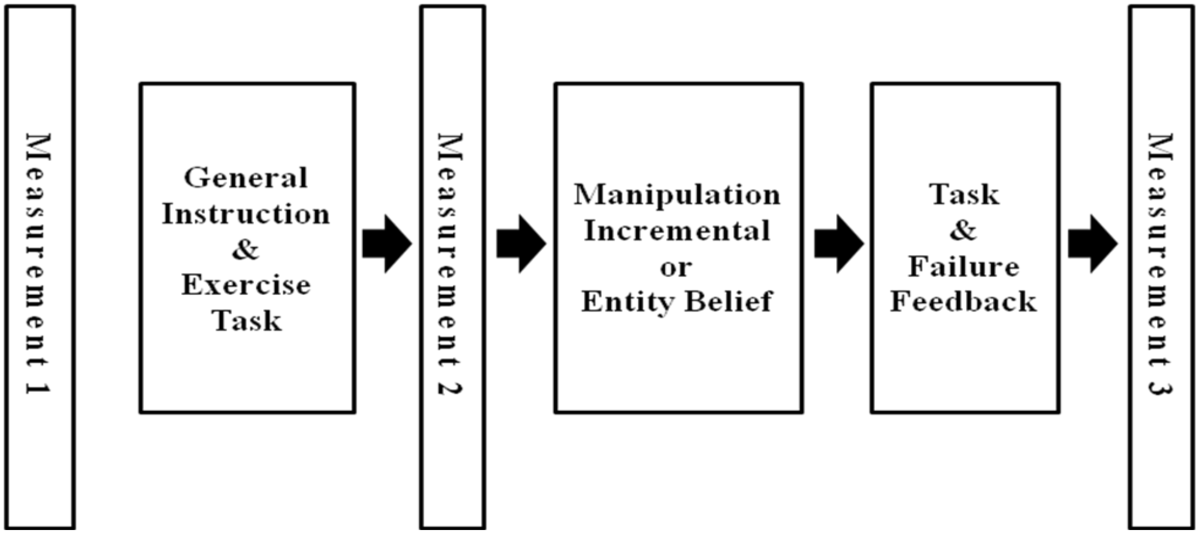

Supplementary Figure 1. Experimental procedures: Anxiety was measure at the measurement 1, and self-efficacy was measure at the measurement 2. Controllability attribution and achievement goals were measure at the Measurement 3.

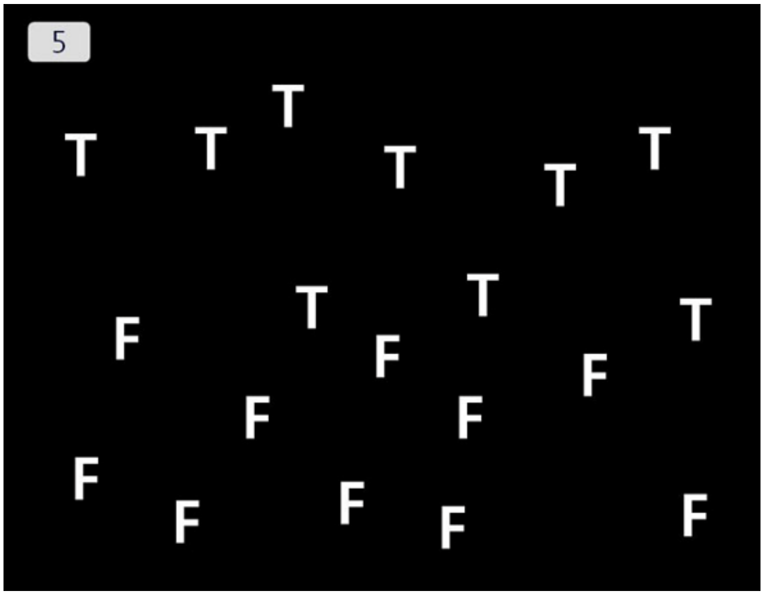

Supplementary Figure 2. Experimental task: A sample trial of the information-processing task.
